# Ezetimibe and atherosclerotic cardiovascular disease: a systematic review and meta-analysis

**DOI:** 10.3389/fcvm.2023.1269172

**Published:** 2023-11-24

**Authors:** Fatemeh Omidi, Maryam Rahmannia, Amir Hashem Shahidi Bonjar, Parsa Mohammadsharifi, Mohammad Javad Nasiri, Tala Sarmastzadeh

**Affiliations:** ^1^ Department of Cardiology, Imam Hossein Hospital, Shahid Beheshti University of Medical Sciences, Tehran, Iran; ^2^ School of Medicine, Shahid Beheshti University of Medical Sciences, Tehran, Iran; ^3^ Clinician Scientist of Dental Materials and Restorative Dentistry, School of Dentistry, Shahid Beheshti University of Medical Sciences, Tehran, Iran; ^4^ School of Pharmacy, Iran University of Medical Sciences, Tehran, Iran

**Keywords:** low-density lipoprotein cholesterol, ezetimibe, systematic review, cardiovascular disease, meta-analysis

## Abstract

**Introduction:**

Individuals diagnosed with atherosclerotic cardiovascular disease (ACD) are exposed to an increased risk of cardiovascular events. Reducing low-density lipoprotein cholesterol (LDL-C) levels has been established as an effective approach to mitigate these risks. However, a comprehensive and up-to-date meta-analysis investigating the LDL-C-lowering effectiveness and the impact on coronary atherosclerotic plaque compositions of Ezetimibe has been lacking.

**Methods:**

We conducted a systematic review by meticulously analyzing databases such as MEDLINE, EMBASE, and the Cochrane CENTRAL for randomized controlled trials that evaluated the efficacy of ezetimibe in lowering LDL-C levels and its influence on coronary atherosclerotic plaques among individuals with ACD. This review encompassed studies available until August 1, 2023. In our analysis, we employed the weighted mean difference (WMD) as the aggregated statistical measure, accompanied by the corresponding 95% confidence interval (CI).

**Results:**

We encompassed a total of 20 eligible studies. Our findings unveiled that the combined therapy involving ezetimibe alongside statins led to a more substantial absolute decrease in LDL-C in comparison to using statins alone. This difference in means amounted to (−14.06 mg/dl; 95% CI −18.0 to −10.0; *p* = 0.0001). Furthermore, upon conducting subgroup analyses, it became evident that the intervention strategies proved effective in diminishing the volume of dense calcification (DC) in contrast to the control group.

**Conclusions:**

Our study findings indicate that the inclusion of ezetimibe in conjunction with statin therapy leads to a modest yet meaningful additional reduction in LDL-C levels when compared to using statins in isolation. Importantly, the introduction of ezetimibe resulted in a significant decrease in the volume of DC. However, it is worth noting that further investigation is warranted to delve deeper into this phenomenon.

## Introduction

Atherosclerotic cardiovascular disease (ACD) stands as the foremost contributor to global mortality. Elevated levels of low-density lipoprotein cholesterol (LDL-C) predominantly underlie the development of ACD (1, 2). To prevent future cardiovascular events, it is highly recommended to focus on the development of novel drugs that target and effectively lower lipoprotein levels. The use of tolerated statins is recommended for reducing LDL-C in patients with ACD. However, it is important to note that even with statin therapy, additional lipid-lowering therapies may be necessary for many patients with clinical ACD. Ezetimibe, a new cholesterol absorption inhibitor drug, has demonstrated the ability to further lower LDL-C levels (3–5). In a randomized controlled trial, it was demonstrated that the combination of ezetimibe with statins significantly reduces levels of LDL-C (6). Additionally, the combination of ezetimibe and statins leads to a substantial decrease in coronary plaque volume compared to statin treatment alone (7). Although the effectiveness of ezetimibe in treating ACD has been acknowledged, there is an absence of a comprehensive and up-to-date meta-analysis regarding the efficacy of ezetimibe in LDL-C lowering in ACD patients. Therefore, this study aimed to systematically assess the efficacy of ezetimibe in reducing LDL-C levels among individuals diagnosed with ACD.

## Methods

### Search strategy

We performed an extensive exploration of PubMed/MEDLINE, EMBASE, and the Cochrane CENTRAL databases to identify studies available until August 1, 2023, that reported on the efficacy and effectiveness of ezetimibe in patients with ACD. The search terms used were ezetimibe, coronary artery, atherosclerosis, and randomized controlled trial. Only studies published in English were included. This study adhered to the PRISMA statement for its design and reporting (Prospero pending ID: 449721) (8).

### Study selection

The collected records from the database searches were merged, and duplicates were removed through the utilization of EndNote X7 (Thomson Reuters, Toronto, ON, Canada). Two reviewers, MR and MJN, conducted a thorough assessment of the records individually, utilizing the title/abstract and full-text screening process to exclude any studies that did not align with the study's objectives.

The studies included in the analysis met the following criteria:

*Participants:* The studies encompassed individuals diagnosed with ACD.

*Intervention:* The intervention being investigated was the use of ezetimibe therapy, either as a standalone treatment or in combination with other lipid-lowering therapies.

*Comparison:* patients who received standard treatment or in combination with other lipid-lowering therapies.

*Outcome:* The primary outcome was the average change observed in LDL-C levels when compared to the baseline measurements.

### Data extraction

Two reviewers, namely MR and MJN, collaboratively devised a structured data extraction form and proceeded to extract information from all qualifying studies. Discrepancies were addressed through mutual agreement. The extracted data encompassed several aspects, including the primary author's name, publication year, study duration, study type, geographical location(s) of the study, ACD patient count, patient age, treatment regimens, demographic details (comprising age, gender, and nationality), and treatment outcomes.

### Quality assessment

The quality assessment of the included studies was conducted by two reviewers (MR and PM) using the Cochrane tool (9). In case of any discrepancies, a third reviewer (MJN) was involved. This assessment tool encompasses several domains, encompassing random sequence generation, allocation concealment, blinding of participants and personnel, blinding of outcome assessors, completeness of outcome data, as well as additional factors like selective reporting and potential biases. Each study underwent categorization based on bias risk: low risk of bias when no bias concerns were detected, high risk of bias when bias concerns were present, or unclear risk of bias when there was an insufficient amount of information available for evaluation.

### Data analysis

The statistical analysis was performed using Comprehensive Meta-Analysis software, version 3.0 (Biostat Inc., Englewood, NJ, USA). The weighted mean difference (WMD) was used as the pooled statistic, with a corresponding 95% confidence interval (CI). The degree of heterogeneity among the studies was assessed using the *I*^2^ value and *p*-value. In cases where the statistical heterogeneity between the studies was low (*I*^2^ ≤ 50% or *p* ≥ 0.1), the fixed-effect model was utilized. Conversely, if a significant level of inter-study heterogeneity was observed (*I*^2^ > 50% or *p* < 0.1), the random-effects model was employed. Cochran's Q test and the *I*^2^ statistic were used to assess between-study heterogeneity. Funnel plots, Egger's and Begg's tests were performed to access the publication bias of studies.

## Results

**Figure 1:**
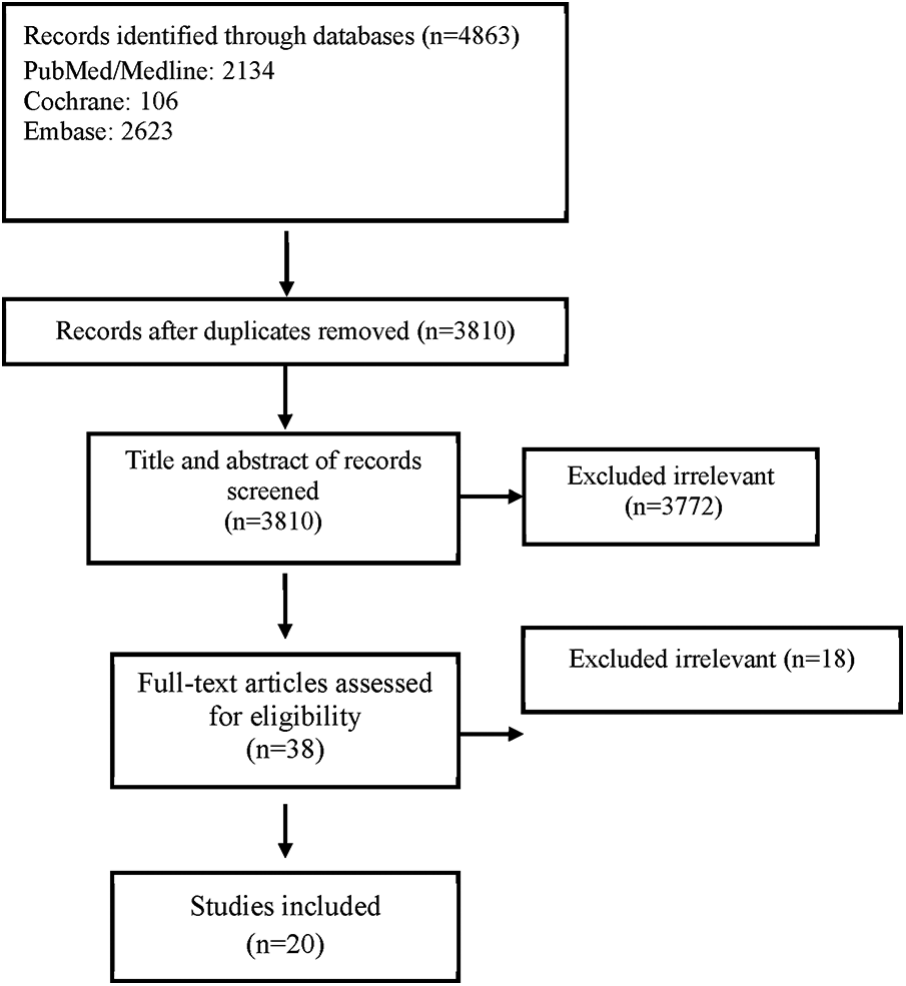
Flow chart of study selection for inclusion in the systematic review and meta-analysis.

Figure 1 illustrates the flow diagram of the systematic review process. This thorough review yielded a total of 20 records that met the specified eligibility criteria. These records reported the primary outcome, which included variations in LDL-C levels from baseline or the LDL-C level after the study, or both. Importantly, these records contained sufficient independent information that enabled the subsequent meta-analysis.

The rationale for including or excluding records is succinctly summarized, and the details of the 20 studies incorporated into the meta-analysis are presented in Table 1. This table provides comprehensive insights into various aspects, such as study design, duration, and baseline demographic characteristics.

**Table 1:** Demographics and baseline characteristics of patients included in the meta-analysis according to study and treatment group.

Authors	Study design	Sample size T/C	Age T	Intervention T	Control C	Duration	% of male
Hougaard (10)	RCT	43/44	55.3 ± 11.0	EZ (10) + AT (80)	PL (10) + AT (80)	12 months	T (90.7) C (81.8)
Jung (11)	RCT	34/36	60.9 ± 10.9	EZ (10) + SI (40)	PR (20)	03 months	T (79.4) C (75)
Kovarnik (12)	RCT	42/47	63.5 ± 9.3	EZ (10) + AT (80)	AT (10)	12 months	T (78.6) C (66)
Brohet et al. (13)	RCT	208/210	63.6 ± 11.1	EZE 10 mg QD + SIM 10/20 mg QD	SIM 10/20 mg QD	6 weeks	T (69.7) C (75.2)
Cannon et al. (14)	RCT	9,067/9,077	63.6 ± 9.7	EZE 10 mg QD + SIM 40–80 mg QD	IM 40–80 mg QD	6 years	T (75.5) C (75.9)
Hibi et al. (15)	RCT	50/53	63 ± 10.0	EZE 10 mg QD + PITA 2 mg QD	PITA 2 mg QD	10 months	T (82) C (77)
Joshi et al. (16)	RCT	40/40	60.3 ± 9.8	EZE 10 mg QD + ROSU 10 mg QD	ROSU 10 mg QD	24 weeks	T (55) C (62.5)
Masuda et al. (17)	RCT	21/19	64.0 ± 7.9	EZE 10 mg QD + ROSU 5 mg QD	ROSU 5 mg QD	6 months	T (90.5) C (84.2)
Ran et al. (18)	RCT	42/42	60.4 ± 8.2	EZE 10 mg QD + ROSU 10 mg QD	ROSU 10 mg QD	10 weeks	T (76.2) C (73.8)
Ren et al. (19)	RCT	55/58	57.3 ± 1.5	EZE 10 mg QD + ROSU 10 mg QD	ROSU 10 mg QD	12 months	T (87.3) C (79.3)
Ueda et al. (20)	RCT	54/54	71 ± 8.0	EZE 10 mg QD + ATOR 10–20 mg QD	ATOR 10–20 mg QD	9 months	T (76) C (81)
Wang et al. (21)	RCT	51/49	58 ± 10.0	EZE 10 mg QD + ATOR 20 mg QD	ATOR 20 mg QD	12 months	T (60) C (61)
Wang et al. (22)	RCT	50/48	63 ± 10.0	EZE 10 mg QD + ROSU 10 mg QD	ROSU 10 mg QD	12 months	T (72) C (73)
West et al. (23)	RCT	18/33	62 ± 8.0	EZE 10 mg QD + SIM 40 mg QD	EZE 10 mg QD	2 years	T (56) C (48)
Zou (24)	RCT	40/40	69.3 ± 5.8	EZE 10 mg QD + ATOR 10 mg QD	ATOR 10 mg QD	12 months	T (
El-Tamalawy et al. (25)	RCT	33/32	61 ± 7.1	EZE 10 mg/day + ATOR 40 mg daily	ATOR 80 mg daily	3 months	T (55) C (70)
Klassen et al. (26)	RCT	10/10	62 (59–64)	EZE 10 mg QD + ROSU 20 mg QD or SIM 40 mg	ROSU 20 mg QD	1 month	T (80) C (70)
Oh et al. (27)	RCT	18/19	56.3 ± 7.1	ATO10 mg + EZE10 mg	ATO 40 mg	12 months	T (72) C (79)
Pinto et al. (28)	RCT	50/51	59 (52–65)	EZE 10mg + SIM 40 mg	ROSU 20 mg	1 month	T (76) C (72)
Blom et al. (29)	RCT	63/73	55.9 ± 9.0	EZE 10 mg + ATOR 80 mg	ATOR 80 mg	1–2 months	T (52.4) C (45.2)

T, treatment group; C, control group; EZ, ezetimibe; ATOR, atorvastatin; SIM, simvastatin.

It is worth noting that while the intended focus was to assess the impact of ezetimibe therapy on LDL-C reduction, either alone or in combination with other lipid-lowering therapies, all the studies included in the analysis contrasted the combined treatment of ezetimibe and statin with statin monotherapy. Participant age across these studies ranged from 57 to 71 years on average, with variations depending on the specific treatment group (Table 1).

### Quality assessment

Regarding the risk of bias across the 20 trials, insufficient detail was provided about the randomization methodology, resulting in an unclear risk of selection bias for random sequence generation and allocation concealment. Other biases were assessed as low in the trials (Table 2).

**Table 2:** Quality assessment.

Author	Random sequence generation	Allocation concealment	Blinding of participants and personnel	Blinding of outcome assessment	Incomplete outcome data
Brohet et al. (13)	L	U	L	U	L
Cannon et al. (14)	U	U	U	U	L
Hibi et al. (15)	L	U	H	L	L
Joshi et al. (16)	L	U	U	U	L
Masuda et al. (17)	L	L	H	H	L
Ran et al. (18)	L	U	H	U	L
Ren et al. (19)	L	U	U	U	L
Ueda et al. (20)	L	U	H	L	L
Wang et al. (21)	U	U	U	U	L
Wang et al. (22)	L	U	U	U	L
West et al. (23)	L	U	L	L	L
Zou (24)	U	U	U	U	L
El-Tamalawy et al. (25)	L	U	L	L	L
Klassen et al. (26)	L	U	L	L	L
Oh et al. (27)	L	U	L	L	L
Pinto et al. (28)	L	U	L	L	L
Blom et al. (29)	L	U	L	L	L
Mikkle et al. (10)	L	U	L	L	L
Jung et al. (11)	L	U	L	L	L
Kovarnik et al. (12)	L	H	L	L	L

L, low; H, high; U, unclear.

### Efficacy of LDL-C lowering of ezetimibe

**Figure 2:**
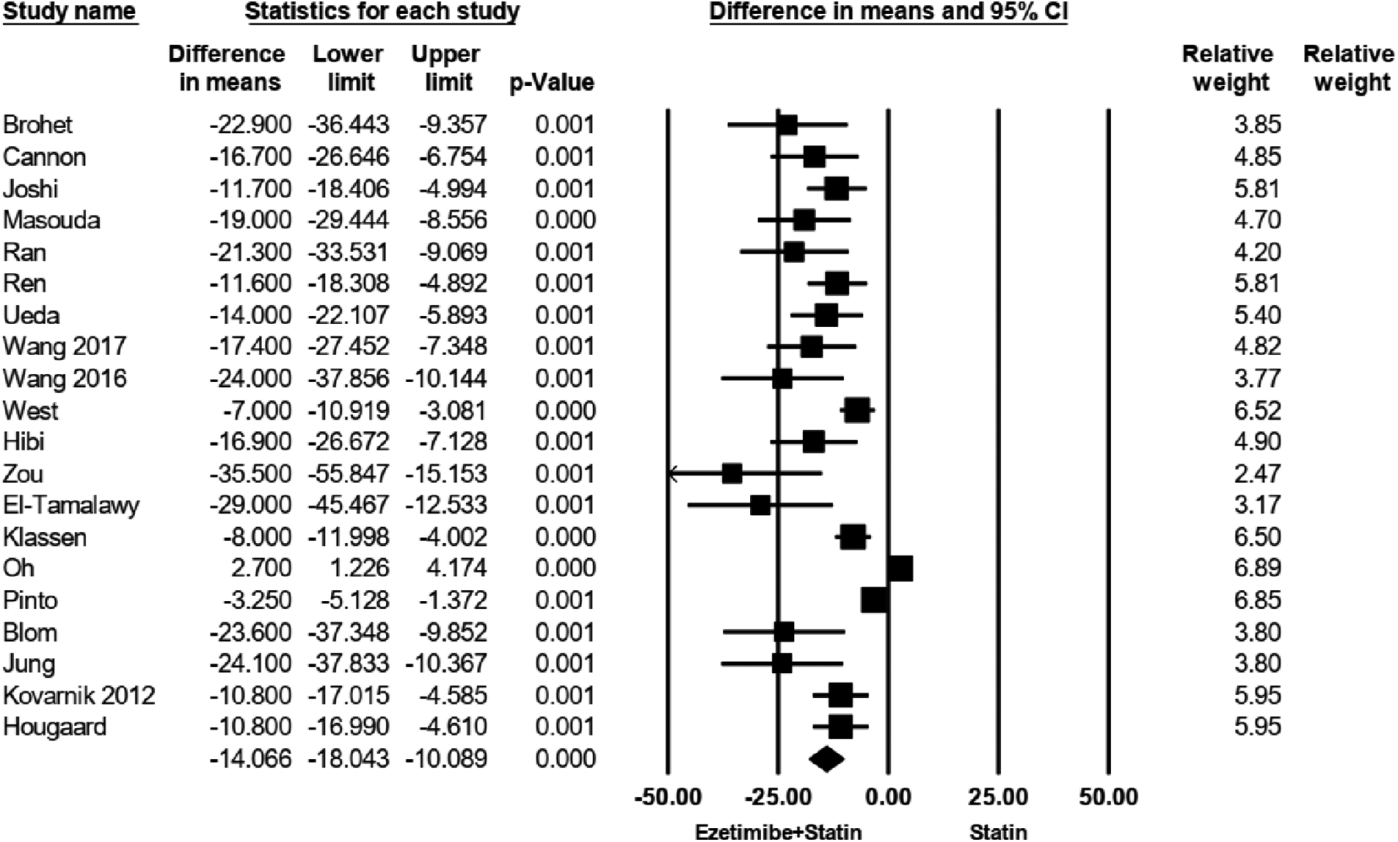
Treatment difference in mean LDL-C change (mg/dl).

As indicated in Figure 2, patients receiving a combination of statin and ezetimibe were found to have a significant additional reduction in LDL-C (WMD = −14.06 mg/dl; 95% CI −18.0 to −10.0, *p *< 0.0001) than those receiving statin alone. As shown in Supplementary Figure S1, some evidence for publication bias was observed (*P* = 0.02 for Begg rank correlation analysis; *P* = 0.03 for Egger weighted regression analysis).

### Fibro-fatty plaque (FFP) volume

**Figure 3:**
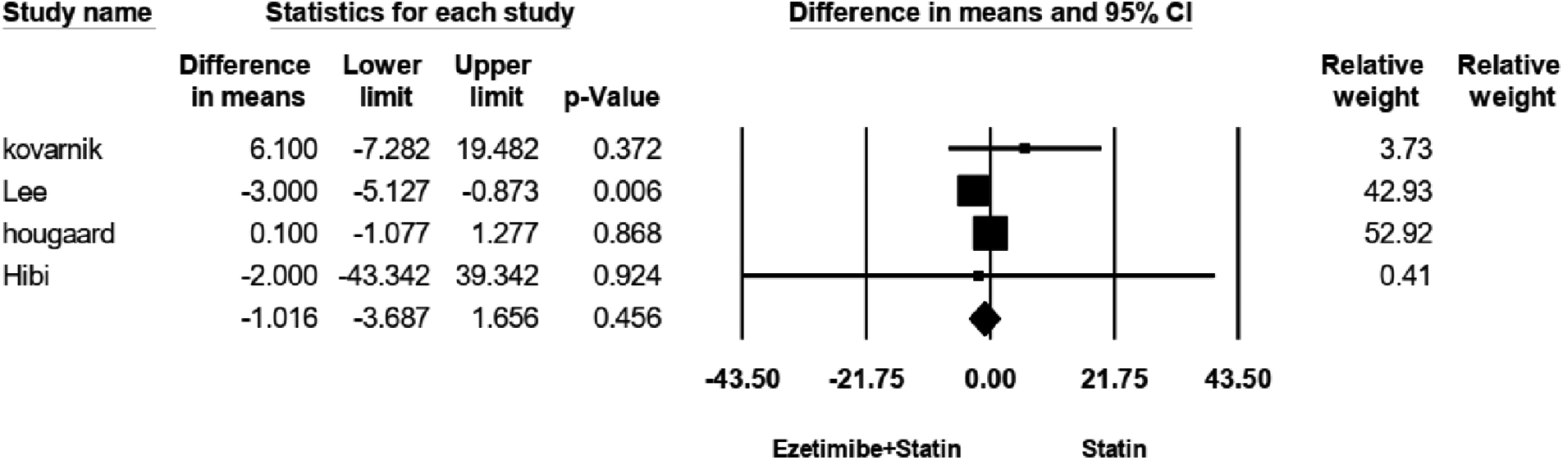
The forest plot for fibro-fatty plaque (FFP) volume.

The efficacy of FFP was evaluated in four studies. No significant heterogeneity was observed among the studies (*I*^2^ = 58.4%, *p* = 0.065). Using a random-effects model for analysis, our findings revealed that the treatment interventions within the study group did not result in a significant reduction in FFP volume when compared to the control group [WMD = −1.01, 95% CI −3.6–1.6, *p* = 0.45], as illustrated in Figure 3. As shown in Supplementary Figure S2, no evidence for publication bias was observed (*P* = 0.2 for Begg rank correlation analysis; *P* = 0.4 for Egger weighted regression analysis).

### Necrotic core (Nc) volume

**Figure 4:**
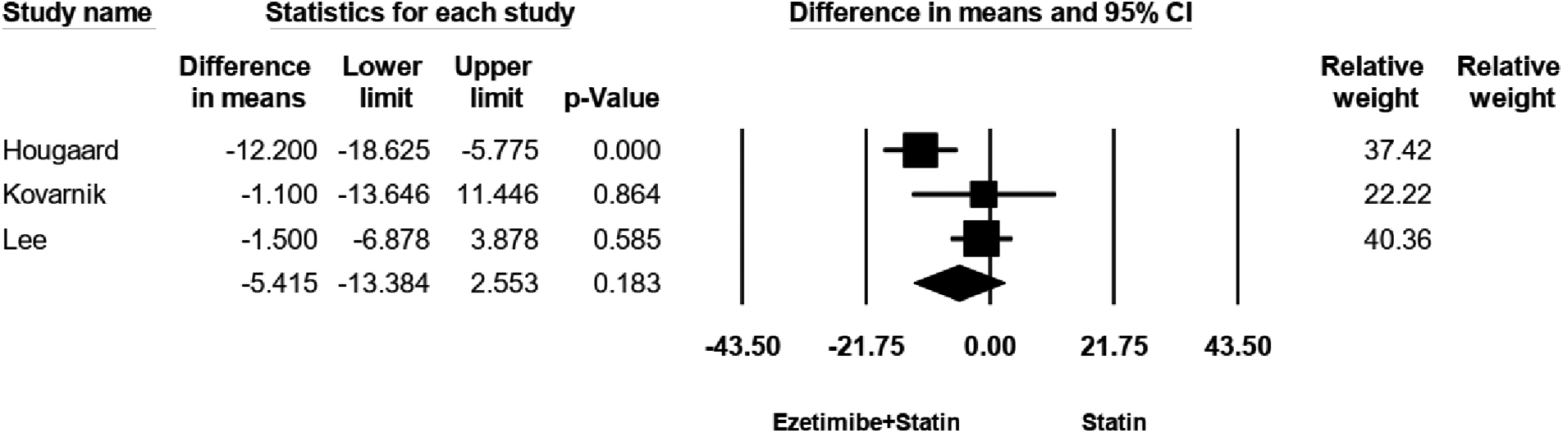
The forest plot for necrotic core (NC) volume.

The effectiveness of NC was documented in three studies. Notably, there was no significant heterogeneity observed among these studies (*I*^2^ = 70.4%, *p* = 0.03). Employing a random-effects model analysis, the outcomes revealed no substantial disparity in the reduction of NC between the treatment group and the control group. The WMD equated to −5.41, with a 95% CI spanning from −13.3 to 2.5, yielding a *p*-value of 0.18, as depicted in Figure 4. As shown in Supplementary Figure S3, no evidence for publication bias was observed (*P* = 0.1 for Begg rank correlation analysis; *P* = 0.5 for Egger weighted regression analysis).

### Change dense calcification (Dc) volume

**Figure 5:**
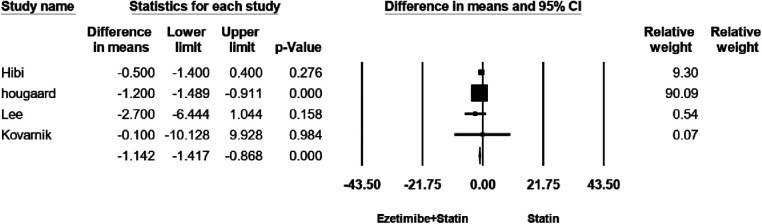
The forest plot for dense calcification (DC) volume.

The efficacy of change DC was assessed in four studies. There was no notable heterogeneity observed among these studies (*I*^2^ = 0%, *p* = 0.42). Employing a fixed-effects model analysis, the findings demonstrated a significant distinction in the reduction of change DC between the treatment group and the control group. The WMD amounted to −1.14, accompanied by a 95% CI ranging from −1.4 to −0.8, resulting in a *p*-value of 0.00, as presented in Figure 5. As shown in Supplementary Figure S4, no evidence for publication bias was observed (*P* = 0.3 for Begg rank correlation analysis; *P* = 0.4 for Egger weighted regression analysis).

## Discussion

### Principal findings

This meta-analysis has showcased that the inclusion of ezetimibe alongside statin therapy yields an extra reduction in LDL-C levels when dealing with patients diagnosed with ACD, in contrast to using statin therapy alone.

### Comparisons with other studies

Our findings align with existing evidence concerning lipid-lowering treatments. For instance, a previous meta-analysis conducted by Shaya et al. also indicated a more significant relative reduction in LDL-C levels with the incorporation of ezetimibe alongside statin therapy, as opposed to using statins alone (30). Another meta-analysis demonstrated that ezetimibe significantly decreased coronary atherosclerotic plaque compared to the control group (placebo or statin monotherapy) (31). Similarly, the research conducted by Toyota et al. corroborates the notion that all three approaches aimed at enhancing LDL-C reduction, namely intensifying statin therapy, incorporating ezetimibe, and introducing PCSK9 inhibitors, have the potential to enhance clinical outcomes among individuals with high atherosclerotic cardiovascular disease (32). Strilchuk et al. also highlights the effectiveness of combining rosuvastatin and ezetimibe for treating hypercholesterolemia and mixed dyslipidemia (33).

### Strengths and limitations of this study

The outcome of our study is in line with the 2020 meta-analysis by Shaya et al., which demonstrated a reduced risk of ACD in patients who underwent ezetimibe treatment (30). Nonetheless, our study boasts certain strengths and distinctive aspects. We delved into studies specifically addressing FFP volume, NC volume, and DC volume. To mitigate the potential influence of confounding factors, we excluded studies involving populations with particular comorbidities. Moreover, articles lacking full text were omitted. On the contrary, we incorporated eight additional studies that were not part of the previous analysis. Our calculated WMD of (−14.06, 95% CI 18.0–10.0) was slightly higher than that of Shaya et al. (−21.8, 95% CI −26.5 to −17.1), while maintaining alignment in terms of direction and statistical significance.

The primary limitation of our study lies in its lack of originality, as the data regarding the efficacy of ezetimibe on LDL-C has been well-established and recognized for a considerable period, even documented in international guidelines. Nevertheless, revisiting and updating the scientific evidence can still hold significance. Several other limitations pertain to the studies we incorporated and are detailed as follows:

Firstly, the absence of recent clinical trial studies is notable, with the most recent one dating back to 2021. This underscores the necessity for further investigations with substantial participant populations to provide deeper insights into the subject.

Secondly, the included studies exhibited confounding factors, and due to limited and inconsistent data, we were unable to perform subgroup analyses to address these confounders adequately.

Thirdly, investigating the mortality benefits of ezetimibe, despite its robust LDL-C lowering effects, presents a notable challenge. Unraveling potential survival advantages hinges on various factors, encompassing drug efficacy, competing risks, off-target effects, baseline cardiovascular risk, and the duration of follow-up in the studies. The fourth limitation of our study is the significant variability in the duration of the included studies, potentially leading to uncertainty in the conclusions we have drawn.

### Clinical implications

This comprehensive systematic review delivers essential insights to decision-makers regarding the advantageous impact of ezetimibe on significant cardiovascular outcomes. Notably, the prominent observations highlight the prevalence of moderate to high certainty evidence that supports the effectiveness of ezetimibe in reducing LDL-C levels. Additionally, these agents contribute to the reduction in the volume of DC.

## Conclusions

Our study indicates that the addition of ezetimibe to statin therapy results in a modest yet significant further reduction in LDL-C compared to statin monotherapy. Ezetimibe led to a significant reduction in DC volume; however, there were no statistically significant differences observed for NC, or change FFP volumes.
