# Supplementary material for: Ezetimibe and atherosclerotic cardiovascular disease: a systematic review and meta-analysis
Source: Front Cardiovasc Med. 2023 Nov 24;10:1269172. doi: 10.3389/fcvm.2023.1269172 (PMC10704196; doi:10.3389/fcvm.2023.1269172)
Supplement: Supplementary file 1 [file Datasheet1.docx]

Figure S1. Funnel plots for LDL-C change.

Figure S2. Funnel plots for FFP.


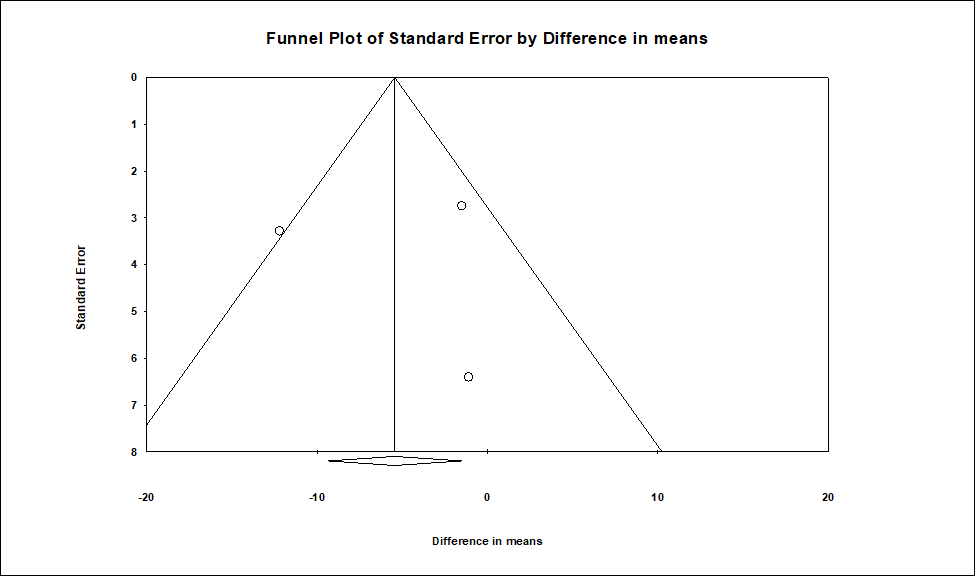


Figure S3. Funnel plots for Necrotic core.


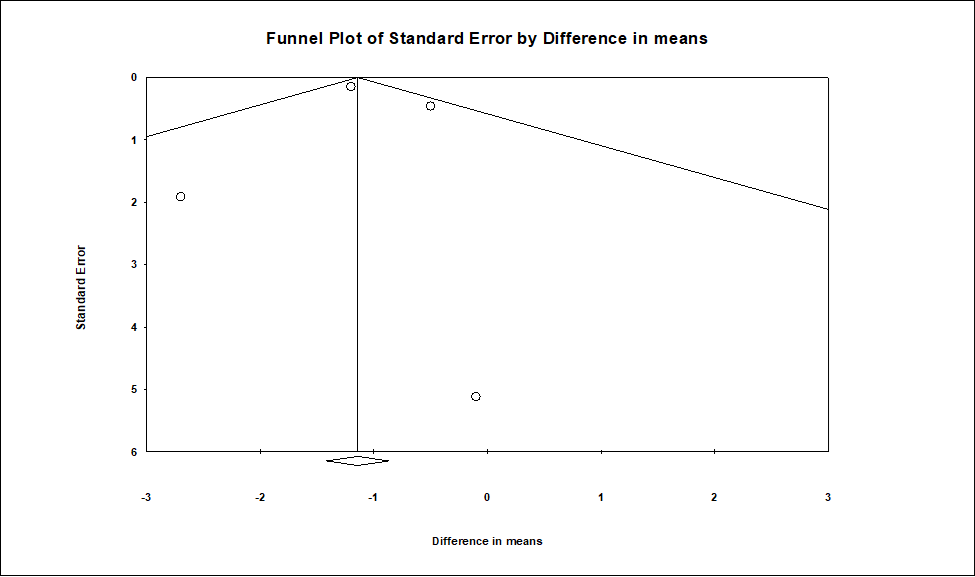


Figure S4. Funnel plots for Dense calcification volume.
